# Cyclocreatine Phosphate: A Novel Bioenergetic/Anti-Inflammatory Drug That Resuscitates Poorly Functioning Hearts and Protects against Development of Heart Failure

**DOI:** 10.3390/ph16030453

**Published:** 2023-03-16

**Authors:** Salwa A. Elgebaly, Charles Van Buren, Robert Todd, Robert Poston, Reem K. Arafa, Nashwa El-Khazragy, Donald Kreutzer, Mostafa A. Rabie, Ahmed F. Mohamed, Lamiaa A. Ahmed, Nesrine S. El Sayed

**Affiliations:** 1 Research & Development, Nour Heart, Inc., Vienna, VA 22180, USA; 2 Department of Surgery, School of Medicine, UConn Health, Farmington, CT 06030, USA; 3 Department of Surgery, Baylor College of Medicine, Houston, TX 77030, USA; 4 Chemistry Department, ProChem Intl., LLC, Sheboygan, WI 53081, USA; 5 Biomedical Sciences Program & Drug Design and Discovery Lab, Zewail City of Science and Technology, Cairo 12578, Egypt; 6 Department of Clinical Pathology-Hematology, Ain Shams Medical Research Institute (MASRI), Faculty of Medicine, Ain Shams University, Cairo 11566, Egypt; 7 Cell & Molecular Tissue Engineering, LLC, Farmington, CT 06030, USA; 8 Department of Pharmacology and Toxicology, Faculty of Pharmacy, Cairo University, Cairo 11562, Egypt

**Keywords:** cyclocreatine phosphate, adenosine triphosphate, anti-inflammatory, heart failure, myocardial ischemia, bioenergetic

## Abstract

Irreversible myocardial injury causes the exhaustion of cellular adenosine triphosphate (ATP) contributing to heart failure (HF). Cyclocreatine phosphate (CCrP) was shown to preserve myocardial ATP during ischemia and maintain cardiac function in various animal models of ischemia/reperfusion. We tested whether CCrP administered prophylactically/therapeutically prevents HF secondary to ischemic injury in an isoproterenol (ISO) rat model. Thirty-nine rats were allocated into five groups: control/saline, control/CCrP, ISO/saline (85 and 170 mg/kg/day s.c. for 2 consecutive days), and ISO/CCrP (0.8 g/kg/day i.p.) either administrated 24 h or 1 h before ISO administration (prophylactic regimen) or 1 h after the last ISO injection (therapeutic regimen) and then daily for 2 weeks. CCrP protected against ISO-induced CK-MB elevation and ECG/ST changes when administered prophylactically or therapeutically. CCrP administered prophylactically decreased heart weight, hs-TnI, TNF-α, TGF-β, and caspase-3, as well as increased EF%, eNOS, and connexin-43, and maintained physical activity. Histology indicated a marked decrease in cardiac remodeling (fibrin and collagen deposition) in the ISO/CCrP rats. Similarly, therapeutically administered CCrP showed normal EF% and physical activity, as well as normal serum levels of hs-TnI and BNP. In conclusion, the bioenergetic/anti-inflammatory CCrP is a promising safe drug against myocardial ischemic sequelae, including HF, promoting its clinical application to salvage poorly functioning hearts.

## 1. Introduction

Heart failure has become an increasingly common problem with a mortality rate higher than most cancers. Part of the difficulty associated with treating HF is its multifactorial and variable pathophysiology. However, a common theme in the pathogenesis of HF is mitochondrial dysfunction with altered bioenergetics and impaired ATP production in the myocardium [1,2,3,4]. Heart muscles require the greatest amounts of energy not only for muscle contraction but also for left ventricle relaxation, which are both highly energy-demanding processes [5]. Cardiac energetics become altered when the mitochondria are unable to supply sufficient ATP to meet demand due to pathologic conditions such as ischemia and hypoperfusion. The cardiomyocyte itself is quickly injured without adequate ATP to maintain cell homeostasis. The lack of high-energy phosphates has multiple pathological downstream harmful events including vascular dysfunction, cardiac inflammation, cytokines, apoptosis, remodeling (fibrosis and collagen deposition), and cardiac dysfunction, which adversely impact patients’ quality of life [6,7,8]. Clearly, there is a major need for therapies that can address this problem both prophylactically and therapeutically.

Although a variety of current therapies have been proven to benefit heart failure patients such as beta-blockers, ACE inhibitors, Aldactone, and Entresto, there are no drugs in development to specifically target abnormal bioenergetics in the creatine phosphate system and their adverse effects on cardiac muscle function in HF patients. Brown et al. [3] indicated that the current treatments for HF patients (beta-blockers, ACE inhibitors, Aldactone, and Entresto) are aimed at developing a reduction in myocardial demand sufficient enough to improve muscle bioenergetics. Our approach, on the other hand, is to improve energy reserves in cardiac muscle under ischemia or hypoperfusion using the synthetic analog of creatine phosphate, CCrP, in order to yield clinically meaningful improvements in muscle function. Accordingly, based on our previous studies [6], CCrP constitutes a new therapeutic strategy by compensating for mitochondrial dysfunction by supplying ATP to restore the adequate ATP levels needed for the appropriate function and homeostasis of cardiac tissues.

CCrP is an FDA orphan drug for heart transplantation, with the designation of “Prevention of Ischemic Injury to Enhance Cardiac Graft Recovery and Survival in Heart Transplantation” (DRU-2015-4951). CCrP is a synthetic analog of the naturally occurring creatine phosphate (CrP). We have previously demonstrated that CCrP is a potent high-energy phosphate donor that is taken up into cardiac tissue and is able to directly phosphorylate adenosine diphosphate (ADP), thereby generating ATP under conditions of limited oxygen supply [6]. CCrP is a long-acting phosphagen with more favorable enzyme kinetics under acidic/anaerobic conditions than CrP [9]. CCrP possesses a substantially less negative Gibbs standard free energy of hydrolysis than CrP and, therefore, it continues to thermodynamically buffer the adenylate system at lower pH values and cytosolic phosphorylation potentials that occur during the later stages of ischemia, conditions in which CrP is no longer effective [9]. Furthermore, Elgebaly SA et al. have previously demonstrated that CCrP preserved high levels of myocardial ATP during ischemia, prevented ischemic injury, reduced cardiac inflammation and apoptosis, and restored normal cardiac function in a variety of animal models of warm and cold ischemia/reperfusion including AMI, global warm cardiac arrest, cardiopulmonary bypass, heart preservation, and heart transplantation [6].

**Figure 1:**
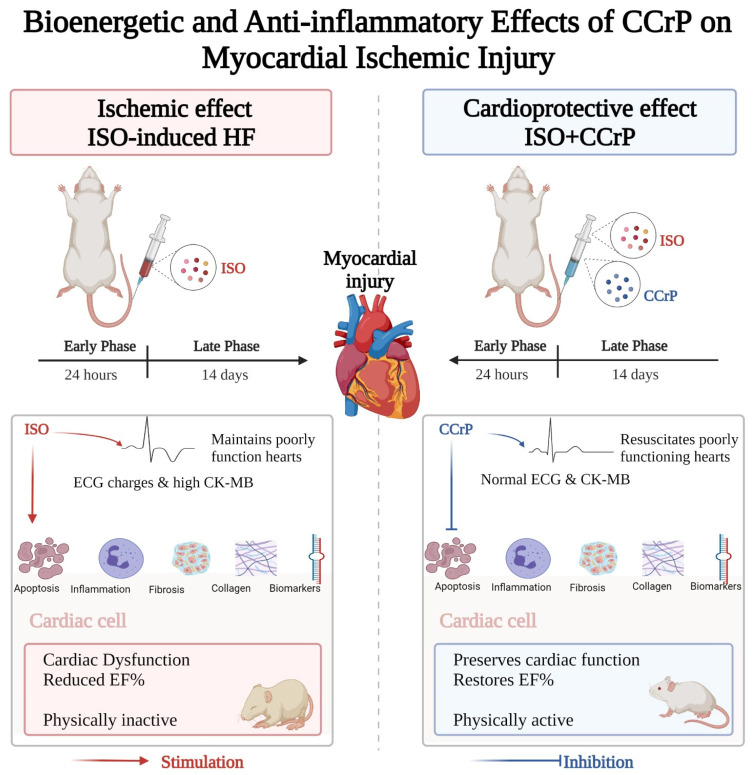
A flow diagram illustrating that the administration of CCrP prevented ischemic injury and resuscitated poorly functioning hearts at the early acute phase of 24 h after the second ISO injection, as shown by normal ECG/ST and CK-MB levels. After 14 days, CCrP prevented the development of HF as indicated by a reduction in apoptosis, inflammation, biomarkers, cardiac remodeling (fibrosis/collagen deposition), and heart weight, with the restoration of the normal ejection fraction, cardiac function, and physical activity in the ISO/CCrP rats. ISO: isoproterenol; CCrP: cyclocreatine phosphate.

The purpose of this study was to evaluate the cardioprotective benefits of CCrP in the standard ISO rat model of ischemia-induced HF [10,11,12,13,14]. We tested the hypothesis that CCrP treatment will prevent ischemic injury and the development of HF when administered prophylactically and will salvage poorly functioning hearts when administered therapeutically. As described in Figure 1, CCrP administered prophylactically (1) prevented ischemic injury in the acute phase of 24 h after the second ISO injection, as shown by normal ECG/ST and CK-MB levels, and (2) significantly protected against the development of HF after 14 days by reducing the downstream harmful events through a marked reduction in cardiac inflammation, apoptosis, biomarkers (hs-TnI, BNP, TNF-α, and TGF-β), remodeling (fibrosis/collagen deposition), and heart weight, but increased expression of eNOS and the cardiac conduction and function, connexin-43 β-actin with the restoration of the normal ejection fraction, cardiac function, and physical activity. When administered therapeutically, CCrP resuscitates poorly functioning hearts in the acute phase of 24 h after the second ISO injection, and the observed normal cardiac function was sustained for an additional 14 days, with the restoration of normal physical activity.

## 2. Results

The results indicated that CCrP administration prevents ischemic injury and the development of heart failure, as well as salvages poorly functioning hearts in the standard ISO rat model of ischemia-induced stress cardiomyopathy. CCrP cardioprotection was observed in both the early acute phase during the first 24 h and the late phase after 14 days. As detailed below, ECG analysis and the levels of serum creatine kinase-MB (CK-MB) (U/L) were conducted 24 h after the second ISO injection. After 14 days, the following analyses were performed: ECG, echocardiography (ECHO) for EF%, heart weight, fibrosis and collagen deposition, cardiac biomarkers and protein expressions (hs-TnI, BNP, TNF-α, TGF-β, caspase-3, eNOS, and connexin-43 β-actin), and physical activity. Data were expressed as the mean ± standard error of the mean (mean ± S.E.M) of three to six rats per group.

### 2.1. CCrP Prevents Acute Myocardial Ischemic Injury

**Figure 2:**
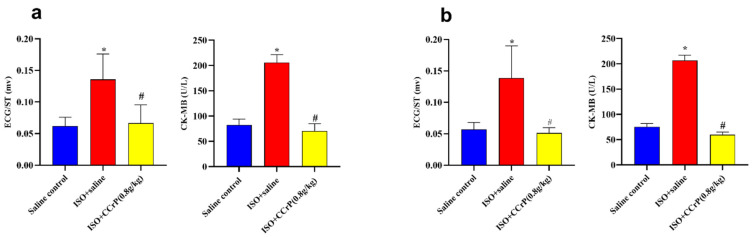
(**a**): CCrP prophylactically administered in rats before the first ISO injection prevents myocardial ischemic injury and cardiac dysfunction measured 24 h after the second ISO injection. (**b**): CCrP therapeutically administered in rats after the second ISO injection salvages poorly functioning hearts. Data are presented as mean ± S.E.M. of 4–6 rats. *: statistical significance (*p* < 0.05) compared to the saline/control group. #: statistical significance (*p* < 0.05) compared to the ISO + saline group. ISO: isoproterenol; CCrP: cyclocreatine phosphate.

Evidence of myocardial injury was documented in the ISO/saline rats 24 h after the second ISO injection via the detection of a high elevation of CK-MB levels (2.78-fold, *p* < 0.05) and ECG/ST changes (2.70-fold, *p* < 0.05) compared to the saline/control group. Results demonstrate that CCrP treatment both prophylactically (Figure 2a) and therapeutically (Figure 2b) prevented myocardial ischemic injury, as indicated by the normalized ST segment together with the CK-MB level compared to those detected in the ISO/saline rats (*p* < 0.05).

### 2.2. CCrP Prevents ECG Perturbations

After 14 days, ISO administration in the ISO/saline rats of both prophylactic and therapeutic regimens induced a significant increase in conduction and contraction abnormalities compared to the saline/control group. Table 1 summarizes the increase in the QT interval (1.88-fold and 1.53-fold, respectively, *p* < 0.05) and QRS duration measurements (1.52-fold and 1.56-fold, respectively, *p* < 0.05), together with a significant decrease in heart rate (79% and 81.7%, respectively, *p* < 0.05). Conversely, significant protection was seen in the ISO/CCrP rats when administered prophylactically or therapeutically with values comparable to the saline/control group (Table 1). Thus, CCrP administration succeeded to normalize electrocardiographic perturbations and heart rate.

### 2.3. Prophylactically Administered CCrP Reduces Inflammation and Remodeling, and Restores Normal Cardiac Function

#### 2.3.1. CCrP Prevents Cardiac Dysfunction and an Increase in Heart Weight Index (HWI)

**Figure 3:**
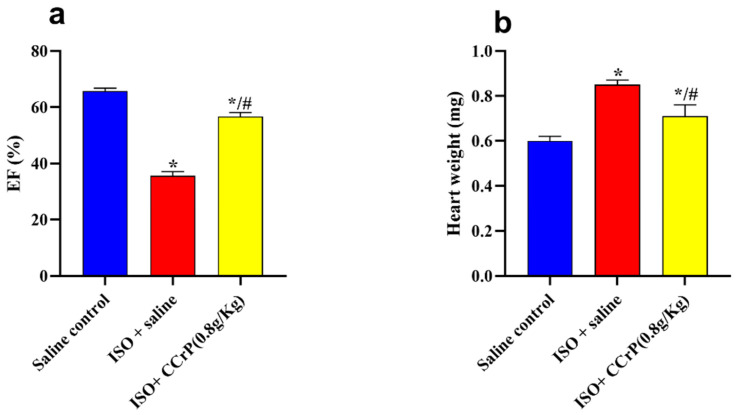
CCrP prophylactically administered before the first ISO injection continued to prevent myocardial injury and the development of cardiac dysfunction (EF%) (**a**) and HWI (**b**) at the end of the 14-day treatment. Data are presented as mean ± S.E.M. of 4–6 rats. *: statistical significance (*p* < 0.05) compared to the saline/control group. #: statistical significance (*p* < 0.05) compared to the ISO + saline group. ISO: isoproterenol; CCrP: cyclocreatine phosphate; and EF: ejection fraction.

Cardiac function (EF%)—after 14 days, ISO/saline rats showed a significant drop in EF% (35.57% ± 2.25) compared to the saline/control rats (63.87% ± 0.29) (*p* < 0.05) (Figure 3a). On the other hand, rats in the ISO/CCrP group showed normal EF% of 63.67% ± 0.13 at the dose of 0.8 g/kg/day, compared to the ISO + saline group (35.57% ± 2.25) (*p* < 0.05). A similar restoration of normal EF% was seen in the ISO/CCrP rats that received CCrP at doses of 0.4 g/kg/day (53.93 ± 0.77) and 1.2 g/kg/day (59.43 ± 1.43) (*p* < 0.05). Furthermore, the administration of CCrP to healthy rats at a dose of 0.8 g/kg/day for 14 days showed a normal EF% of 61.57% ± 0.64 (*p* < 0.05), suggesting a lack of heart toxicity.

HWI—ISO/saline rats showed a significant increase in HWI (0.85 ± 0.02 mg) compared to the saline/control rats (0.60 ± 0.02 mg) (1.32-fold, *p* < 0.05), indicating myocardial hypertrophy (Figure 3b). On the other hand, ISO/CCrP rats at 0.8 g/kg/day had a reduced HWI by 83.25% of that of the ISO/saline rats (*p* < 0.05) (Figure 3b). Additionally, CCrP treatment at 0.4 and 1.2 g/kg/day showed a reduction in HWI by 78.88% and 80.09%, respectively.

**Figure 4:**
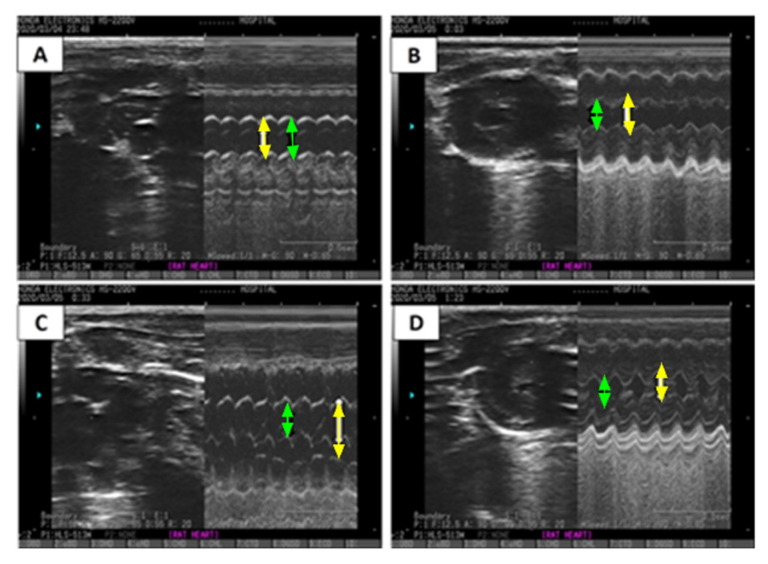
ECHO images demonstrate the effect of CCrP on ISO-induced changes in M-mode in LVEDD (yellow arrow) and LVESD (green arrow). Groups include saline/control (**A**), CCrP/control (**B**), ISO/saline (**C**), and ISO + CCrP (**D**). CCrP at a dose of 0.8 g/kg/day. ISO: isoproterenol; CCrP: cyclocreatine phosphate; LVEDD: left ventricular end-diastolic diameter; and LVESD: left ventricular end-systolic diameter.

Additionally, an ECHO analysis was performed for verification, and images are presented in Table 2 and Figure 4. Figure 4 reveals a significant increase in left ventricular end-diastolic diameter (LVEDD) (1.30-fold) (*p* < 0.05), left ventricular end-systolic diameter (LVESD) (1.66-fold) (*p* < 0.05), and E/A ratio in the ISO-induced HF rat model (Table 2 and Figure 4C) compared to the saline/control rats (Table 2 and Figure 4A). Treatment with CCrP at a dose of 0.8 g/kg/day in the ISO-induced HF model reverted LVEDD and LVESD to normal conditions (Table 2, Figure 4C,D), with EF% values of 63.67% ± 0.13 comparable to the saline/control rats (Figure 3a). Furthermore, healthy rats that received CCrP at a dose of 0.8 g/kg/day for 14 days showed normal LVEDD (7.93 ± 0.03) and LVESD (5.53 ± 0.09) comparable to those seen in the saline/control rats, suggesting a lack o-*f heart toxicity due to CCrP administration (Figure 4B).

#### 2.3.2. CCrP Reduces Cardiac Inflammation and Remodeling (Fibrin and Collagen Deposition)

**Figure 5:**
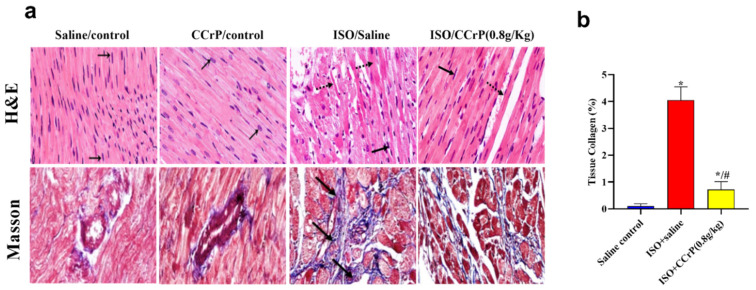
(**a**) Histopathological analysis of H&E (cardiac inflammation) and Masson’s trichrome-stained (cardiac fibrin and collagen deposition) heart sections related to the effect of different doses of CCrP on myocardial fibrosis associated with ISO administration (black arrows—area of fibrosis). Specimens stained with Masson’s trichrome for estimation of myocardial fibrosis (blue color) in saline/control, CCrP/control, ISO/saline, and ISO/CCrP rats at a dose of 0.8 g/kg/day. (**b**) ISO/CCrP rats at a dose of 0.8 g/kg showed an 83% reduction in collagen% compared to levels detected in the ISO/saline rats. Data are presented as mean ± S.E.M. (n = 5.) *: statistical significance (*p* < 0.05) compared to the saline/control group. #: statistical significance (*p* < 0.05) compared to the ISO + saline group. ISO: isoproterenol; CCrP: cyclocreatine phosphate.

Cardiac inflammation and remodeling—histopathological analysis of hematoxylin–eosin (H&E) and Masson’s trichrome-stained heart sections was conducted by two independent pathologists blinded to the various groups. Results indicated the detection of a marked cardiac inflammatory response and an intense increase in fibrin and collagen deposition in the ISO/saline rats (score: +2 to +3), which were not seen in the ISO/CCrP rats at the dose of 0.8 g/kg/day (score: 0 to +1) (Figure 5a). Analysis further showed extensive fibrous deposition in the ISO/saline rats, while the ISO/CCrP rats showed delicate fibrous tissue between the myocardial bundles, almost close to normal. The quantitative percentage of tissue collagen deposition is presented in Figure 5b. The ISO/saline rats showed a marked increase in collagen deposition (4.05-fold) (*p* < 0.05), while CCrP at a dose of 0.8 g/kg/day showed strong protection by inhibiting collagen deposition by 83% (Figure 5b). At a dose of 1.2 g/kg/day, collagen deposition was inhibited by 94%. Fibrin and collagen deposition were undetectable in the saline/control rats (Figure 5a,b). Similarly, both fibrin and collagen deposition were undetectable in the CCrP control rats where healthy rats received CCrP at a dose of 0.8 g/kg/day for 14 days, suggesting a lack of heart toxicity due to CCrP administration.

#### 2.3.3. CCrP Restores Cardiac Biomarkers and Protein Expression

**Figure 6:**
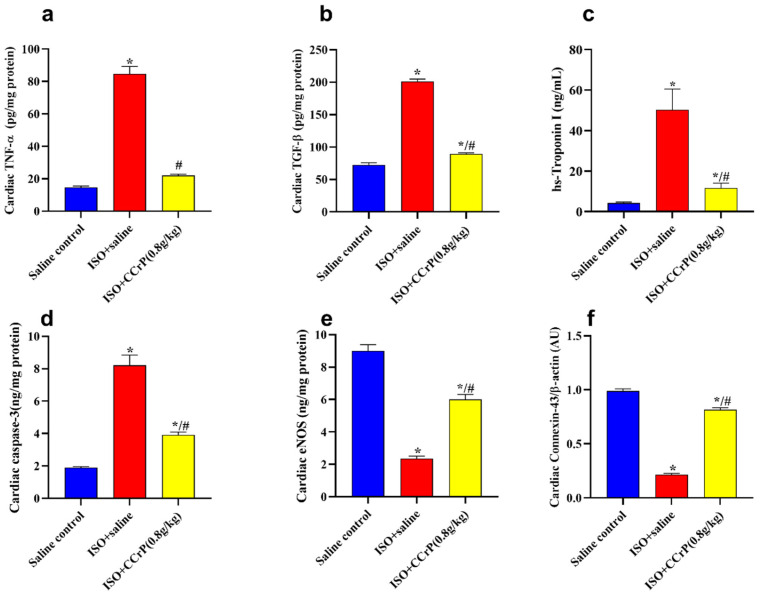
(**a**) TNF-α: ISO/CCrP rats given a dose of 0.8 g/kg/day showed a significant reduction in TNF-α compared to the ISO/saline rats; (**b**) TGF-β: ISO/CCrP rats showed a significant reduction in TGF-β compared to the ISO/saline rats; (**c**) Tn I: ISO/CCrP rats showed a significant reduction in Tn I compared to the ISO/saline rats; (**d**) Caspase-3: ISO/CCrP rats showed a significant reduction in Caspase-3 contents compared to the ISO/saline rats; (**e**) eNOS: ISO/CCrP rats showed a significant increase in eNOS contents compared to the ISO/saline rats; and (**f**) connexin-43 β-actin: ISO/CCrP rats showed a significant increase in connexin-43 β-actin contents compared to the ISO/saline rats. Data are presented as mean ± S.E.M. of 4–6 rats. *: statistical significance (*p* < 0.05) compared to the saline/control group. #: statistical significance (*p* < 0.05) compared to the ISO + saline group. ISO: isoproterenol; CCrP: cyclocreatine phosphate; TNF-α: tumor necrosis factor-alpha; TGF-β: transforming growth factor-beta; and eNOS: endothelial nitric oxide synthase.

Cardiac biomarkers and protein expression—the ISO/saline rats showed a marked elevation in TNF-α, TGF-β, hs-cTnI, and caspase-3 contents and a reduction in eNOS and the cardiac conduction and function, connexin-43 β-actin (Figure 6). Isoproterenol-treated rats showed a substantial elevation in cardiac TNF-α (4.2-fold) (Figure 6a), TGF-β (2.9-fold) (Figure 6b), hs-cTnI (16.6-fold) (Figure 6c), and caspase-3 contents (5.4-fold) (Figure 6d) compared to the saline/control rats, indicating the stimulation of the fibrotic and inflammatory pathways, as well as myocardial injury and apoptosis. Such a rise was markedly mitigated (50% to 80%) by CCrP treatment at the effective dose of 0.8 g/kg/day when compared to the levels detected in the ISO–saline rats (Figure 6). Additionally, ISO/saline rats showed a marked decline in cardiac eNOS contents (32.8%) (Figure 6e) and connexin-43 β-actin expression (21.8%) (Figure 6f). CCrP treatment at a dose of 0.8 g/kg/day markedly enhanced the expression of cardiac eNOS (2.74-fold) and connexin-43 β-actin contents (3.74-fold) compared to the ISO/saline rats. Furthermore, CCrP treatment at doses of 0.4 g/kg/day and 1.2 g/kg/day significantly decreased the levels of TNF-α, TGF-β, and hs-cTnI and caspase-3 contents by up to 80%, and increased the expression of cardiac eNOS (up to 2.38-fold) and connexin-43 β-actin contents (up to 3.41-fold) when compared to the ISO/saline rats. Similarly to the saline/control group, the CCrP/control rats showed normal expression levels of TNF-α, TGF-β, hs-cTnI, and caspase-3 contents, as well as cardiac eNOS and connexin-43 β-actin contents, further suggesting a lack of heart toxicity due to CCrP administration.

### 2.4. Therapeutically Administered CCrP Salvages Poor Heart Function and Sustains Normal Cardiac Function

**Figure 7:**
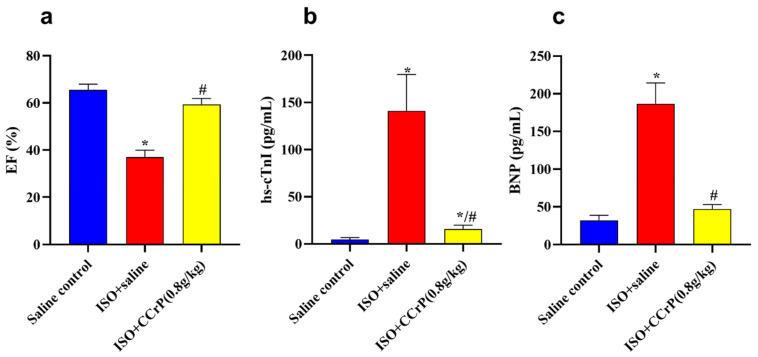
(**a**) ISO/saline showed a significant reduction in EF%, which was restored to normal levels due to the therapeutic treatment of CCrP at the dose of 0.8 g/kg/day; CCrP treatment also significantly reduced levels of TnI (**b**) and BNP (**c**). Data are presented as mean ± S.E.M. of 4–6 rats. *: statistical significance (*p* < 0.05) compared to the saline control group. #: statistical significance (*p* < 0.05) compared to the ISO + saline group. ISO: isoproterenol; CCrP: cyclocreatine phosphate; EF: ejection fraction; hs-cTnI: high-sensitivity Troponin I type 3; and BNP: brain natriuretic peptide.

Cardiac function (EF%)—therapeutically administered CCrP at a dose of 0.8 g/kg/day showed quick restoration of poorly functioning hearts in rats with a high elevation of CK-MB and ECG/ST changes observed in the acute phase after 24 h after the second ISO injection (Figure 3). The observed preservation of heart function in the ISO/CCrP rats was sustained over the long term of an additional 14 days, as indicated by normal EF% (56.68 ± 1.42%) compared to the significantly dropped EF% in the ISO/saline rats (35.58 ± 1.53%) (*p* < 0.05) (Figure 7a). The saline/control rats showed a normal EF% of 65.70 ± 1.07% (Figure 7a).

CCrP treatment at 0.8 g/kg/day also mitigated the high serum levels of hs-cTnI (pg/mL) in the ISO/saline rats (148 ± 11.16) (36.9-fold) compared to the saline control (4.71 ± 0.74) (*p* < 0.05) rats, where the ISO/CCrP rats showed reduced levels of hs-TnI (21.08 ± 2.99) (*p* < 0.05) (Figure 7b). Similarly, the ISO/saline rats showed elevated serum levels of BNP (pg/mL) (185 ± 10.39) (5.7-fold) compared to the saline control rats (32.45 ± 2.32), where CCrP treatment at 0.8g/kg/day showed reduced levels of hs-BNP (57.71 ± 4.11) (*p* < 0.05) (Figure 7c). The marked rise in serum levels of hs-cTnI and BNP confirms the presence of cardiac dysfunction and HF (Figure 7c). Both increases were significantly hampered by CCrP treatment, suggesting the ability of CCrP to salvage poorly functioning hearts from stress cardiomyopathy both in the acute and late phases of the disease.

### 2.5. CCrP Restores Normal Physical Activity

**Figure 8:**
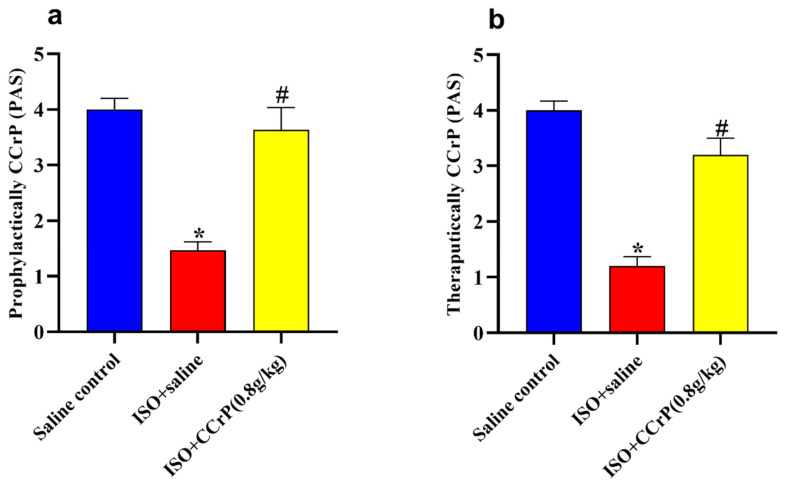
Treatment of CCrP at the dose of 0.8 g/kg/day restores normal physical activity in both prophylactic (**a**) and therapeutic (**b**) regimens. Data are presented as mean ± S.E.M. of 4–6 rats. *: statistical significance (*p* < 0.05) compared to the control/saline group, #: statistical significance (*p* < 0.05) compared to the ISO + saline group. ISO: isoproterenol; CCrP: cyclocreatine phosphate; PAS: physical activity score.

After 14 days, the ISO/saline rats showed low physical activity scores in both prophylactically (Figure 8a) and therapeutically (Figure 8b) administered CCrP, while the ISO/CCrP rats showed normal physical activity.

## 3. Discussion

CCrP is a synthetic analog of CrP which is a potent high-energy phosphate donor that is taken up into the heart tissue and is able to directly phosphorylate adenosine diphosphate, thereby generating ATP under conditions of limited oxygen supply [6,9]. It has a longer half-life and more favorable enzyme kinetics under acidic/anaerobic conditions than creatine phosphate [9]. We have demonstrated that CCrP administration improves myocardial energetics by preserving high levels of myocardial ATP during ischemia and restoring cardiac function in a variety of animal models of ischemia/reperfusion including AMI, global warm cardiac arrest, cardiopulmonary bypass, heart preservation, and heart transplantation [6].

There is growing recognition that the next generation of therapeutics for HF must address the problem of bioenergetic failure [3]. Currently, there are two ways to improve ATP stores in cardiomyopathy: reduce utilization or increase supply. Efforts to reduce ATP utilization in the myocyte of cardiomyopathy patients using Mavacamten or Ranolazine have shown mixed results [15,16]. Elamipretide is a cell-permeable peptide that enhances mitochondrial ATP production through the interaction with cardiolipin to promote electron transportation. Recently approved anti-hyperglycemic drugs, sodium–glucose co-transporter-2 (SGLT2) inhibitors, improve mitochondrial efficiency by favoring B-hydroxybutyrate oxidation of the heart over fatty acids. This improves the efficiency of ATP production under conditions of limited oxygen supply. Elamipretide and SCLT2 inhibitors showed successful results for preserving mitochondrial function in pre-clinical studies and in patients with heart failure and reduced ejection fraction [3]. However, their effect on HF is still undergoing trials. Another target for the treatment of HF is the abnormal cardiac remodeling and increasing of stiffness and fibrosis that characterize this disorder. Alterations in intracellular nitrogen mono-oxide signal cascade secondary to oxidative stress are key steps in this process. Vericiguat is an oral soluble guanylate cyclase stimulator that directly generates cyclic guanosine monophosphate and restores the sensitivity of soluble guanylate cyclase to endogenous nitric oxide. Despite the promising primary trials that showed a favorable effect of the drug in improving physical activity when administered to HF patients, large RCTs failed to confirm these findings [17].

No prior studies have addressed the problem of inefficient production of ATP within the myocytes by directly increasing ATP supply via the phosphocreatine system. Additionally, the therapeutic benefits of CCrP in animal models of heart failure have not been reported. Therefore, based on our previous studies [6], we believe that the ability of CCrP to preserve high levels of cellular ATP with anti-inflammatory properties makes it a promising novel target for early therapeutic intervention in those with HF. The advantage of CCrP over other therapies designed for this purpose is the simplicity of its mechanism; it merely augments a natural physiological process via CrP so that more ATP is available under conditions where the normal means of supplying ATP are unable to meet demand. Unlike any other drug in development, CCrP does not interfere with normal metabolism. No other agents proposed to treat HF work through a similar mechanism of effect on the phosphocreatine system. Instead, other therapies that attempt to improve ATP supply do so by altering the mitochondrial function.

In the present study, we used the standard ISO rat model of ischemia-induced HF [13,14] and demonstrated that the prophylactic administration of CCrP at the effective dose of 0.8 g/kg/day protected hearts against ischemic injury in the acute phase of 24 h after the last ISO injection, and prevented the subsequent development of HF after 14 days. Similarly, CCrP administered therapeutically after the completion of the second ISO injection resuscitated poorly functioning hearts in the acute phase and continued to preserve normal cardiac function and physical activity for an additional 14 days. Specifically, CCrP significantly altered the underlying mechanism involved in the pathogenesis of HF by (1) maintaining normal heart weight, cardiac function (EF%), and normal physical activity; (2) resuscitating poorly functioning hearts with sustained preservation of normal physical activity after 14 days; (3) significantly reducing the levels of cardiac biomarkers including hs-TnI, BNP, TNF-α, TGF-β, and caspase-3 contents; (4) markedly inhibiting cardiac inflammation and apoptosis; (5) significantly increasing the levels of eNOS and connexin-43 β-actin; and (6) markedly reducing cardiac remodeling (fibrosis and collagen deposition) by over 83%. Furthermore, CCrP at a lower dose of 0.4 g/kg/day and a higher dose of 1.2 g/kg/day continued to show cardioprotection. Thus, the administration of CCrP significantly protected hearts against ischemic injury and the development of HF, as well as resuscitated poorly functioning hearts by reducing the number of downstream harmful events, including cardiac inflammation, apoptosis, and remodeling resulting in the restoration of normal cardiac function and the physical activity of rats. These results support our hypothesis that CCrP treatment prevents ischemic injury and the development of HF when administered prophylactically, and salvages poorly functioning hearts when administered therapeutically.

Previously, we have reported that CCrP treatment inhibited a cardiac molecular mechanism involved in the pathogenesis of HF via the autophagy-related Nourin-dependent *miR-137* (a marker of myocardial ischemic damage which promotes cardiac remodeling in HF) and Nourin-dependent *miR-106b* (a marker of inflammation which promotes cardiac inflammation) [12]. Specifically, the Nourin-associated miR-137 and miR-106b were significantly upregulated in the ISO/saline rats, but not in the ISO/CCrP rats that did not develop HF [12], further supporting the anti-inflammatory property of CCrP.

The results of the limited safety study, where healthy rats were treated with CCrP at a dose of 0.8 g/kg/day for 14 consecutive days (CCrP control), showed no toxicity in rat hearts as evidenced by the absence of cardiac inflammation, apoptosis, biomarkers, and remodeling, and that these rats continued to exhibit normal cardiac function and physical activity similar to the saline control rats. The lack of cardiac toxicity further confirms our recently reported studies that the administration of CCrP did not alter liver and renal function, suggesting that CCrP is a safe drug [12].

We believe that CCrP can potentially have a number of clinical applications as a bioenergetic/anti-inflammatory cardioprotective drug in heart transplantation, high-risk cardiac surgery, intervention cardiology, delayed AMI patients, and patients with Takotsubo cardiomyopathy.

A. Heart transplantation—The number of heart transplants performed each year in the U.S. has significantly lagged behind liver and kidney placement, with 3902 heart transplants in 2021 compared to 8896 liver transplants and 23,401 kidney transplants [18,19]. In this regard, the percentage of hearts donated in 2021 was only 28% of organ donors as 6 hearts from every 10 organ donors are unutilized. This happens due to the following two important factors: (1) donors with poor heart function, and (2) ischemic injury caused by harvesting and ex vivo cold static storage, particularly transport periods that exceed 4 h. As an FDA orphan designated drug for the “Prevention of Ischemic Injury to Enhance Cardiac Graft Recovery and Survival in Heart Transplantation” (DRU-2015-4951), CCrP can improve donor heart bioenergetics which will allow for prolonged transport times (e.g., >6 h compared to the current 4 h) [6] and salvage poorly functioning donors’ hearts. Thus, CCrP can potentially increase donor heart utilization and the safety of heart transplantation beyond what is possible with current methods.

B. High-risk cardiac surgery and intervention cardiology—CCrP can potentially protect against complications in the following areas:

(1) Patients who have completed the high-risk transcatheter aortic valve implantation (TAVI) procedure. Significant complications of high-risk TAVI procedures include death (6–7% one-year mortality), heart failure (19–23%), and stroke (up to 5%) [20]. During the procedure, myocardial ischemia occurs, which can cause low cardiac output syndrome (LCOS) at the end of the procedure. We believe that CCrP administration immediately prior to the TAVI procedure will significantly protect hearts against this initial ischemic injury and have a positive impact on reducing short- and long-term complications triggered by LCOS.

(2) Patients who are undergoing high-risk cardiac surgery. There are approximately 200,000 cardiac surgical procedures performed per year in the U.S. About 10% of these cases are considered to be high-risk, defined as more than 5% predicted risk of perioperative mortality. This risk can be accurately predicted based on calculations performed prior to surgery that depend on the procedure, comorbidities, and other factors available in the medical record. The main reason that high-risk patients die after surgery is due to LCOS. This clinical syndrome is often characterized by reduced cardiac ejection fraction after surgery, poor cardiac output relative to needs, hypotension, and acidosis, and it eventually leads to multiorgan failure and death. Much of this problem results from poor cardiac protection during the period of cardiac arrest required to perform the surgical procedure. Efforts to improve this protection, using CCrP, may improve cardiac function after surgery and reduce the mortality associated with LCOS.

(3) Patients who are undergoing intervention cardiology. There are approximately one million percutaneous coronary intervention (PCI) procedures performed per year in the U.S. About 10% of these procedures are considered to be high-risk. Protecting cardiac tissues during these high-risk cases will potentially reduce cardiac injury and preserve cardiac function.

C. Delayed AMI patients—Every year, about 805,000 people in the U.S. experience AMI [21]. Currently, more than 50% of patients with AMI seek medical care late [21]. A delay after the onset of infarction in treatment using current existing strategies leads to a higher incidence of heart failure (HF), particularly for women [22,23]. Based on our positive results that CCrP resuscitates poorly functioning hearts and preserves prolonged normal cardiac function, we believe that CCrP administration to patients with delayed AMI will potentially protect adjacent areas from progressing to necrotic myocardium and reduce the subsequent development of HF.

D. Takotsubo cardiomyopathy—This refers to the treatment of patients with Takotsubo cardiomyopathy to reduce death or congestive heart failure. Takotsubo cardiomyopathy (approximately 25,000 cases yearly) is due to ischemia-induced non-ST elevation myocardial infarction (non-STEMI) thought to be associated with acute physical or psychological stress. The disorder is not due to coronary artery disease and is characterized by a typical apical ballooning of the left ventricle shown via echocardiogram that usually improves over the course of several months. However, stress cardiomyopathy can lead to death or congestive heart failure with 80 to 90% of cases being women [24,25]. A registry has been established to record these cases and distinguish them from STEMI cases [26]. Of note, the abnormal echocardiography findings and proposed pathophysiology in Takotsubo cardiomyopathy patients are comparable to those observed in organ donors with poor cardiac contractility [27]. Since this cardiomyopathy is associated with acute cardiac stress, and we have demonstrated in the present study that CCrP resuscitates poorly functioning hearts, maintains prolonged normal cardiac function, and prevents the development of HF in the ischemia-induced myocardial injury rat model of Takotsubo, it appears reasonable that the acute treatment of Takotsubo patients with CCrP might speed up recovery and minimize the long-term morbidity and/or mortality from this disorder.

In conclusion, CCrP is a promising novel bioenergetic/anti-inflammatory drug that prevents ischemic injury and the development of HF by blocking key harmful downstream events of ischemia (inflammation, apoptosis, remodeling, and cardiac dysfunction). Potential future therapeutic applications of CCrP include heart transplantation, high-risk cardiac surgery, intervention cardiology, patients with Takotsubo cardiomyopathy, and patients with delayed AMI.

## 4. Materials and Methods

### 4.1. Experimental ISO Rat Model (ISO/Saline “HF Rats” and ISO/CCrP “Non-HF Rats”)

A standard ISO rat model of ischemia-induced heart failure was used to determine whether CCrP administered prophylactically prevents ischemic injury and the subsequent development of heart failure (HF) and whether the administration of CCrP therapeutically resuscitates poorly functioning hearts and sustains the preserved cardiac function over 14 days. We purchased from Cairo University Research Park’s Animal Technology Laboratory (Dokki, Cairo, Egypt) adult male Wistar rats (6–8 weeks old) weighing 180–220 g. Rats were caged under controlled temperature (20–25 °C) and humidity (45–55%) conditions with a twelve hour light/dark cycle and free access to food and water. Prior to the experiment, animals were housed for a couple of weeks to accommodate them, and then they were randomly distributed across the groups. Solutions of highly pure CCrP (>98%) (Nour Heart, Inc., Vienna, VA, USA) were freshly prepared before use.

All experimental procedures were approved by the Faculty of Pharmacy Ethics Committee at Cairo University (permit number: PT 2733), and they were carried out in accordance with the US National Institutes of Health’s Guide for Care and Use of Laboratory Animals (NIH publication no. 85-23, revised in 2011). The project began on 19 May 2019.

#### 4.1.1. CCrP Administered Prophylactically

Rats were injected with isoproterenol hydrochloride (Sigma Aldrich; Merck KGaA, Darmstadt, Germany, Cat. # 15627) subcutaneously (s.c.) for two consecutive days at specific doses of 85 and 170 mg/kg/day (the LD50 for s.c. isoproterenol in rats = 600 mg/kg), respectively, and then left for an additional two weeks [10,11,12,13,14]. The ISO/saline rats were treated with 1 mL saline via intraperitoneal (IP) injection both 24 h and 1 h before the first ISO administration, and then daily for two weeks. The ISO/CCrP rats were also treated via IP injection with 1 mL CCrP solution 24 h and 1 h before the first ISO administration, and then daily for an additional two weeks. Prior to use, solutions of CCrP were freshly prepared in saline. According to our previous studies, CCrP at a dose of 0.8 gm/kg/day is the most effective dose to prevent myocardial ischemic injury in intact canine and rat models of acute myocardial ischemia, global cardiac arrest, cardiopulmonary bypass, and heart transplantation [6,10,11,12].

Adult male Wistar rats were divided into four groups: (1) ISO/saline (n = 6), where ISO rats were treated with IP injections of saline; (2) ISO/CCrP (n = 5), where ISO rats were treated with IP injections of CCrP at a dose of 0.8 g/kg/day; (3) saline/control (n = 5), where healthy rats were treated with IP injections of saline; and (4) CCrP/control (n = 4), where healthy rats were treated with IP injections of CCrP at a dose of 0.8 g/kg/day for 14 days to evaluate the potential drug toxicity [12]. The cardioprotective activity of CCrP was also tested using a lower dose of 0.4 g/kg/day (n = 3) and a higher dose of 1.2 g/kg/day (n = 2). Additionally, various parameters including serum CK-MB (rat Creatine Kinase MB Isoenzyme ELISA Kit (Cat no: DEIA-FN285) Creative Diagnostics, New York, NY, USA) and ECG analysis were conducted 24 h after the first dose of ISO to confirm the development of early myocardial injury. Fourteen days after the second ISO injection, we measured the following parameters: heart weight, EF%, ECHO analysis, and physical activity, as well as cardiac fibrosis, percentage of deposited collagen, serum levels of high-sensitivity rat cardiac Troponin I, BNP, and various protein expressions in the ventricles including tumor necrosis factor-alpha (TNF-α) (inflammation marker), transforming growth factor-beta (TGF-β) (fibrogenic marker), vascular function-endothelial nitric oxide synthase (eNOS), apoptosis-caspase-3, and the cardiac conduction and function-connexin-43 β-actin.

In humans, CCrP will be used at the effective dose of 0.21 gm/kg for an average 100 kg person [28].

#### 4.1.2. CCrP Administered Therapeutically

For this study, a total of 18 male Wistar albino rats (170 to 190 gm) were used. Isoproterenol hydrochloride was procured from Sigma-Aldrich, St. Louis, MO, USA, Cat. # 15627. ISO was administered subcutaneously to rats over the course of two consecutive days at doses of 85 mg/kg (first day) and 170 mg/kg (second day) [10,11,12,13,14]. Only rats (n = 10) that showed cardiac dysfunction via electrocardiographic abnormalities (ST elevation) and high serum creatine kinase-MB 24 h after the second ISO injection were allowed to proceed to complete the study for an additional 14 days. These 10 rats with poor heart function were randomized into two groups and received saline or CCrP one hour after completing the course of the second ISO injections and then daily for an additional 2 weeks. Four rats received a saline injection (1 mL/i.p.) (ISO + saline, n = 4) and six rats received CCrP at an effective dose of 0.8 g/kg/day (ISO + CCrP, n = 6) [10]. A negative control group of healthy rats was injected with saline (saline/control, n = 4). After 14 days, evidence of stress cardiomyopathy was assessed via ECHO analysis for EF% measurements, serum levels of hs-TnI and BNP, and physical activity.

### 4.2. Assessment of Heart Function

The electrical activity of the heart was measured 24 h and 14 days after the second ISO injection to evaluate the acute and long-term responses to CCrP treatment. An electrode was inserted subcutaneously into the limb under anesthesia (50 mg/kg of thiopental) to record ECGs. The ECGs were used to calculate heart rate, QT interval, and QRS duration. After 14 days, heart function was evaluated using ECHO, which used an ultrasound probe (Honda HS-2200 V, Tokyo, Japan) to measure the dimensions of the left ventricle and calculate the ejection fraction percentage. This was achieved using a 12.5-MHz probe, and the measurements were taken as an average over three cardiac cycles. The LVEDD and LVESD, as well as the EF%, were automatically calculated in the M-Mode of the long-axis parasternal view and provided by the built-in software [11].

### 4.3. Physical Activity

Fourteen days after the second ISO injection, the scoring of physical activity was recorded as follows: normal activity (+4); mild physical impairment (+3); moderate physical impairment (+2); and severe physical impairment (+1) [6].

### 4.4. Sample Processing and Heart Weight Index (HWI) Determination

Twenty-four hours after the last ISO injection, serum samples were collected to measure the levels of CK-MB using the rat creatine kinase MB isoenzyme ELISA Kit (Cat. No. DEIA-FN285; Creative Diagnostics, USA). The instructions provided by the kit’s manufacturer were followed. At the end of the study, 14 days after the last ISO injection, rats were sacrificed via decollation under anesthesia using 30 mg/kg pentobarbital. Hearts were quickly removed and rinsed with cold phosphate-buffered saline and weighed after removing the atria, aorta, and fat. The weights of the rats were also recorded and the heart weight index (HWI, in mg/g) was calculated by dividing heart weight by rat weight. Hearts from 3 rats from each group were used for a histopathological examination. The remaining rats’ ventricles were quickly dissected, rinsed, dried, and weighed. One part was homogenized in cold phosphate-buffered saline to create a 10% homogenate, and the other part was frozen at −80 °C for later use in further analysis.

### 4.5. Biochemical Assessment

Enzyme-linked immunoassay (ELISA) was used to measure brain natriuretic peptide (BNP) (MyBioSource, Inc., San Diego, CA, USA, Cat. # MBS2700198) and rat Troponin I type 3 (cTn-I) in the serum as markers for HF using the rat high-sensitivity Troponin I type 3 (cTn-I) (MyBioSource, Inc., San Diego, CA, USA, Cat. # MBS765393). Additionally, ELISA was used to measure the levels of tumor necrosis factor-alpha (TNF-α) (Cat. # MBS2507393), transforming growth factor-beta (TGF-β) (Cat. # MBS260302), endothelial nitric oxide synthase (eNOS) (Cat. # MBS721860), and caspase-3 (Cat. # MBS763727) in tissue homogenates using corresponding kits (MyBioSource, Inc., San Diego, CA, USA).

Protein concentrations were assessed via Western blot analysis on a portion of the ventricle that was homogenized in lysis buffer. A connexin-43 primary antibody (Thermofisher Scientific, Waltham, MA, USA, Cat. # 71-0700) and horseradish peroxidase (HRP)-conjugated goat anti-rabbit secondary antibody (Thermofisher Scientific, USA, Cat. # 31460) were used to measure protein expression and determine protein levels using a Bicinchoninic acid (BCA) protein assay kit (Thermo Fisher Scientific Inc., USA) [13]. Using a scanning laser densitometer from Biomed Instrument, Clinton Charter Township, MI, USA, the amount of protein was determined via densitometric analysis of the autoradiograms. The results were adjusted to beta-actin and expressed as a change from the normal levels in the saline control group.

### 4.6. Histopathological Assessment

The heart tissues of the rats were preserved in a 10% formalin solution and embedded in paraffin wax blocks. Five-micrometer sections were cut using an ultra-microtome and sections were stained with H&E. The slides were examined under a light microscope (Olympus, Tokyo, Japan) and the images were captured using a digital camera. Additionally, a semi-quantitative scoring system (morphometry) was used to measure the amount of cardiac collagen, with 0 indicating no collagen, 1 indicating moderate collagen, and 3 indicating intense collagen. Sections of the hearts (5 m) were stained with Masson trichrome to evaluate the extent of myocardial fibrosis and to identify collagen fibers in cardiac tissues using an image analyzer (Leica Qwin 550, Wetzlar, Hesse, Germany). The percentage of fibrosis for each group was calculated by calculating the average of 10 randomly selected fields from each section. Sections were also stained with H&E to evaluate myocardial damage and hypertrophy. A semi-quantitative grading scale of 0–5 was used to assess myocardial injury [14]. Two independent pathologists who were blinded to the study groups and the experimental setup examined the heart morphology for each section.

### 4.7. Statistical Analysis

Quantitative values were expressed as the mean ± standard error of the mean (mean ± S.E.M) of 3 to 6 rats per group. The significant difference in the measured variables between different experimental groups was data analyzed using GraphPad Prism (version: 8; GraphPad Software Inc., San Diego, CA, USA). The analysis of variance test (ANOVA) followed by Dunn’s multiple comparison test were used to compare multi-group results. A *p*-value <0.05 was considered to be statistically significant. Normality was checked using the Shapiro–Wilk test and homogeneity was checked using Levene’s test.

## Figures and Tables

**Table 1:** ECG changes associated with prophylactic and therapeutic regimens with CCrP.

	Prophylactically Administered CCrP			Therapeutically Administered CCrP		
	Saline/Control	ISO + Saline	ISO + CCrP (0.8 g/kg/day)	Saline/Control	ISO + Saline	ISO + CCrP (0.8 g/kg/day)
QT (ms)	138.3 ± 4.41	260.0 ± 11.55 *	160.0 ± 5.77 ^#^	156.70 ± 8.8	240.0 ± 5.7 *	170.7 ± 15.72 ^#^
QRS (ms)	35.33 ± 1.76	53.67 ± 4.48 *	36.67 ± 2.90 ^#^	38.0 ± 1.15	59.3 ± 2.40 *	41.33 ± 3.52 ^#^
HR (bpm)	350.0 ± 5.77	277.0 ± 5.87 *	326.3 ± 10.87 ^#^	340.0 ± 5.7	278.3 ± 6.00*	329.3 ± 9.82 ^#^

Data are presented as mean ± S.E.M. of 4–6 rats. *: statistical significance (*p* < 0.05) compared to the saline/control group. ^#^: statistical significance (*p* < 0.05) compared to the ISO + saline group. ANOVA: one-way analysis of variance. ISO: isoproterenol; CCrP: cyclocreatine phosphate; and HR: heart rate.

**Table 2:** After 14 days, ISO/CCrP rats showed normal LVEDD and LVESD, while ISO/saline rats showed a significant increase. * *p* < 0.05 for ISO/saline vs. saline/control rats, and ^#^ *p* < 0.05 for ISO/CCrP vs. ISO/saline rats.

Group	LVEDD (mm)	LVESD (mm)
Saline control	7.77 ± 0.15	5.43 ± 0.03
ISO + saline	10.07 ± 0.12 *	9.00 ± 0.15 *
ISO + CCrP	8.03 ± 0.09 ^#^	6.27 ± 0.07 ^#^

## Data Availability

The data presented in this study are available upon request from the corresponding author.

## Abbreviations

ATP: adenosine triphosphate; ADP: adenosine diphosphate; AMI: acute myocardial infarction; BNP: brain natriuretic peptide; CrP: creatine phosphate; CCr: cyclocreatine; CCrP: cyclocreatine phosphate; CK-MB: creatine kinase-myocardial band; eNOS: endothelial nitric oxide synthase; EF%: ejection fraction percentage; ELISA: enzyme-linked immunoassay; ECHO: echocardiography; ECG: electrocardiographic; HF: heart failure; HWI: heart weight index; H&E: hematoxylin and eosin; HRP: horseradish peroxidase; ISO: isoproterenol; LVEDD: left ventricular end-diastolic diameter; LVESD: left ventricular end-systolic diameter; LCOS: low cardiac output syndrome; miR: microRNA; miR-137: microRNA-137; miR-106b: microRNA-106b; mean ± S.E.M: mean ± standard error of mean; STEMI: ST-elevation myocardial infarction; NSTEMI: non-ST elevation myocardial infarction; PCI: percutaneous coronary intervention; TNF-α: tumor necrosis factor-alpha; Tn-I: rat high-sensitivity Troponin I type 3; TGF-β: transforming growth factor-beta; TAVI: transcatheter aortic valve implantation.
